# Nursing home-acquired pneumonia: course and management in the emergency department

**DOI:** 10.1186/1865-1380-7-19

**Published:** 2014-05-12

**Authors:** Syed Imran Ayaz, Nadia Haque, Claire Pearson, Patrick Medado, Duane Robinson, Robert Wahl, Marcus Zervos, Brian J O’Neil

**Affiliations:** 1 Department of Emergency Medicine, Wayne State University School of Medicine, 4201 St. Antoine, 6G-UHC, Detroit, MI 48201, USA; 2 Division of Infectious Diseases, Henry Ford Health System, 2799 West Grand Boulevard, Detroit, MI 48202, USA

**Keywords:** Nursing home, Pneumonia, Emergency department

## Abstract

**Background:**

Pneumonia is among the foremost causes of hospitalization and mortality in patients residing in extended care facilities. Despite its prevalence, there is currently little literature focusing on the course and management of nursing home-acquired pneumonia (NHAP) in the emergency department (ED). Our objective was to investigate the ED presentation, course, management and outcomes in patients admitted through the ED with NHAP.

**Methods:**

A retrospective chart review of nursing home patients with a presumptive or final diagnosis of pneumonia admitted through the ED was performed at two large hospitals in Detroit, Michigan.

**Results:**

A total of 296 patients were included in the study from 2002 to 2007 with a mean age of 81.1 years (SD ± 10.95) and 55.4% females. Blood cultures were performed on 90.8% of patients in the ED; 17.8% of these revealed growths, but half of these were considered contaminants. Initial chest x-ray in the ED was read as possible pneumonia in 18.2% of patients; 73.9% were started on antibiotics (ABX) in the ED. Mean hospital length of stay (LOS) was 10.75 days (SD ± 9.35) and in-hospital mortality was 16.2%. Time until first ABX in univariate analysis was nearly significant (*p* = 0.053) for mortality prediction, and the appropriate versus inappropriate ABX (per the Infectious Diseases Society of America and American Thoracic Society guidelines) did not affect mortality. Patients treated with a single ABX had significantly increased LOS (*p* = 0.0089). There was poor correlation between LOS and time until first ABX as well as LOS and time until appropriate ABX with a correlation coefficient of -0.048 (*p* = 0.42) and -0.08 (*p* = 0.43), respectively.

**Conclusions:**

In this data set of NHAP patients admitted through the ED, we found a surprisingly low prevalence of true-positive blood cultures, high incidence of antibiotic pre-treatment at nursing homes prior to admission, high hospital mortality and low immunization rates. There was a wide spectrum of pathogens grown in blood culture. Only two thirds of the patients had dyspnea at presentation, and less than half had either cough or fever. On physical examination, about one fourth had no clinical findings consistent with pneumonia. Further, less than one fifth of chest x-rays were interpreted as possible pneumonia.

## Background

Pneumonia is among the foremost causes of hospitalization and mortality in patients residing in extended care facilities [1-8]. The microbes causing nursing home-acquired pneumonia (NHAP) are not well defined and include a wide spectrum from pathogens involved in community-acquired pneumonia (CAP) to those specific to nosocomial infections [9,10]. In 2005, the Infectious Diseases Society of America (IDSA) and American Thoracic Society (ATS), based upon data available to them, recommended empiric antibiotic treatment (ABX) guidelines for healthcare-acquired pneumonia (HCAP) including NHAP [11]. These treatment recommendations have not yet realized universal adherence, which may be due in part to the paucity of data available on NHAP [12,13].

It has been well documented that the discrepancy in defining NHAP is an impediment toward a standardized approach to its management [12]. Absence of a precise scoring system for hospitalized patients with NHAP has also led to prognostic models that are inadequate in stratifying disease severity [12]. According to the 2005 ATS/IDSA guidelines, a new or progressive infiltrate seen on chest X-ray plus compatible clinical signs, such as new-onset fever with temperature greater than 38°C, purulent sputum, leukocytosis or hypoxia, are essential features for the clinical diagnosis of HCAP, including NHAP [11].

Latest evidence suggests that transfer of nursing home patients with pneumonia to acute care facilities resulted in minor and insignificant improvement in mortality or morbidity when compared with patients who were treated at the nursing homes [14]. Once admitted, these patients are frequently managed as CAP in the hospital [1]. The management of NHAP continues to be controversial because of the scarcity of information on risk factors and diagnosis specific to this group of patients and lack of randomized controlled clinical trials that could help determine the best antimicrobial treatment regimen, length of treatment and rapid molecular techniques to ascertain etiological agents [15]. A previous study investigated the attitudes of physicians toward NHAP treatment guidelines and found that the core obstacles to implementation of the ATS/IDSA guidelines are apprehensions regarding the practicality of using the suggested regimens and absence of documented improved outcomes [13].

Advances in modern medicine have led to a rapid increase in the aging population with a subsequent increase in the number of elderly population requiring medical care [16]. An increase in the annual incidence of NHAP of up to 1.9 million cases is expected over the next 3 decades [14]. Despite its prevalence, there is currently little literature focusing on the course and management of NHAP in the ED. Similarly, little is known regarding the early predictors of mortality in these patients in the ED. Our objective was to investigate the ED presentation, course, management, outcomes, pathogens, appropriateness of ABX, time to first ABX administration and efficacy of ABX in relation to mortality and length of stay (LOS) as well as early predictors of mortality and LOS in patients admitted through the ED with NHAP. An understanding of the risk factors and outcomes of these patients is critical in the design of prevention and treatment strategies.

## Methods

An Institutional Review Board (IRB)-approved, retrospective chart review was performed at two large tertiary care, academic teaching hospitals in Michigan. Henry Ford Hospital in Detroit is an 800-bed urban hospital with 93,000 emergency department visits annually. Similarly, Beaumont Hospital in Royal Oak is a 1000-bed community hospital with 115,000 emergency department visits per year.

Patients that resided in a nursing home or an extended care facility and who were admitted through the ED with a presumptive or final diagnosis of pneumonia were included in this retrospective chart review study. Patients were excluded from data abstraction if any of the following were noted during data abstraction: active cancer, tuberculosis, HIV, any immune suppression and hospitalization within the 3 weeks prior to the study visit. A standardized chart abstraction was performed collecting patient demographics, laboratory studies, radiographs, comorbidities, LOS, course of treatment, final outcome and ABX (pre- and post-ATS/IDSA guidelines). Our review of ABX included the time that the first antibiotics were ordered and administered and time until appropriate antibiotic administration (per the ATS/IDSA guidelines for HCAP). Additionally, we captured recommended antimicrobial therapy versus single antibiotic treatment on mortality and LOS. The included cases were retrospectively reviewed by trained abstractors using a standardized abstraction form. This study was performed in accordance with the previously published methods of Gilbert and colleagues [17].

All variables were entered into Statistical Package for Social Sciences (SPSS version 17.0^©^ SPSS Inc.) and descriptive analysis was performed. Means and standard deviations were calculated for continuous variables and frequencies determined for categorical variables. Categorical variables were examined using Pearson’s chi-square and Fisher’s exact test where appropriate. Continuous variables were analyzed using a Wilcoxon rank test and Spearman correlation. Step-down multiple-variable logistic regression analyses were performed to determine the strongest predictors of mortality.

## Results

A total of 296 patients were included in the study from 2002 to 2007. Out of these 206 (69.6%) patients were admitted to Beaumont Hospital and 90 (30.4%) patients were admitted to Henry Ford Hospital. The mean age of patients was 81.1 years (SD ± 10.9). The gender distribution was 164 (55.4%) females and 132 (44.6%) males. A total of 237 (80.1%) patients had pneumonia as one of the admission diagnoses. Other admission diagnoses were 20.9% sepsis, 17.2% UTI, 10.1% dehydration, 11.8% CHF, 9.5% acute renal failure/renal insufficiency, 7.1% acute respiratory failure, 6.8% COPD and 4.5% atrial fibrillation; 20.6% of the patients who had an admission diagnosis of pneumonia did not have a discharge diagnosis of pneumonia; 18.9% had received vaccinations in the past 1 year; 11.5% were vaccinated against influenza and 14.9% were vaccinated against pneumococcus. Eighty-two (27.7%) patients had antibiotic therapy initiated in the nursing home (Table 1).

**Table 1:** Comorbidities and antibiotics started at the nursing home

**Comorbidities**	**% of Patients**	**Nursing home antibiotics**	**% of Patients**
Hypertension	82.8	Fluoroquinolones	14.5
Dementia	55.1	Amoxicillin and clavulanate	3.3
CHF	42.2	Co-trimoxazole	3.1
Stroke/TIA	39.2	Macrolides	2.7
COPD	30.7	Cephalosporins	2.2
Renal disease	30.7	Vancomycin	2.0
Diabetes mellitus	26.4	Anti-fungal	2.0
Alzheimer’s	22.3	Piperacillin and tazobactam	1.2
Liver disease	1.7	Nitrofurantoin	1

**Figure 1:**
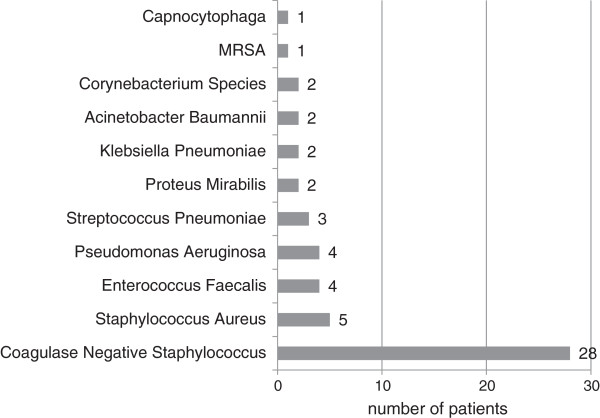
Pathogens revealed in blood culture performed in the ED.

**Figure 2:**
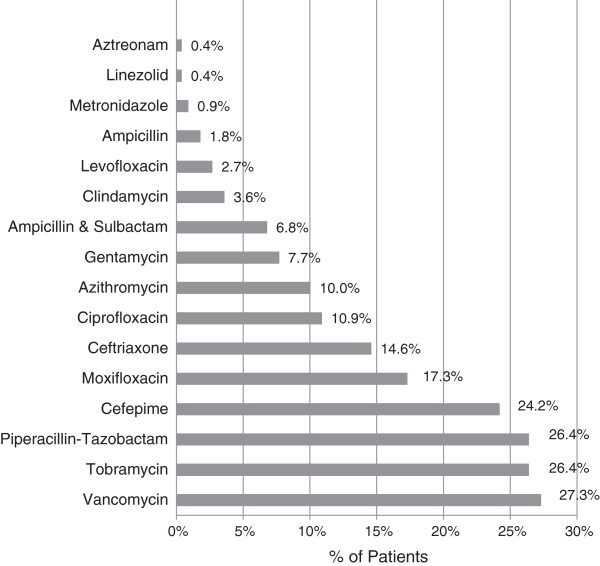
Antibiotic therapy started in the ED.

Blood cultures were performed on 90.87% of patients in the ED, 17.8% of whom revealed growth of organisms; however, 50% of these were considered to be contaminants. Contaminants were either defined as such in the medical record or were coagulase-negative staphylococcus in a non-immunocompromised patient (Figure 1). Sputum culture was obtained in 28% of the patients, 83.1% of which grew bacteria, none of which correlated to the identified blood pathogens. Two hundred nineteen (73.98%) patients were given antibiotics in the ED (Figure 2). Cardiac enzymes were drawn in 75.7% of ED patients and were positive in 13.85%. On initial ED ECG, 25.7% had signs of ischemia and 18.2% of the patients showed signs of pneumonia on their initial ED chest X-ray as interpreted by the final radiologist report.

Of the patients, 41.2% were admitted to the regular medical floor, 19.3% to the telemetry floor, 20.9% to step-down unit and 17.6% to the ICU; 12.16% of the patients were intubated for a mean duration of 9.28 days (SD ± 12.2). Mean hospital LOS was 10.75 days (SD ± 9.35), mean LOS in the ICU was 1.72 days (SD ± 5.1), mean LOS in step-down/progressive care was 2.57 days (SD ± 5.3), and mean LOS on regular medical floor with telemetry services was 6.2 days (SD ± 7.3). Of the patients, 65.5% were discharged back to the nursing home, 3% were discharged to a rehabilitation facility, 9.1% were discharged under hospice care, and 3.7% were discharged home; 16.2% of the patients expired during the initial hospitalization. Twenty-five percent of these were intubated, 25% of these were ICU-admits and 20.96% of were admitted to the step-down unit.

Of the patients, 41.6% were readmitted to the hospital within 1 year of their initial visit. Out of these, 54.5% were discharged back to nursing homes, 3.3% to a rehabilitation facility, 6.5% under hospice care, and 14.6% home, and 8.1% of these patients expired during the 1-year follow-up re-admission to the hospital.

Comparing patients before and after the publication of the 2005 ATS/IDSA guidelines, NHAP was appropriately treated per the guidelines 59.4% versus 41.3%, treated as CAP 26.1% versus 18.7% of the time and treated with a single antibiotic 46.4% versus 33.3%, respectively. All comparisons were statistically significance with a *p*-value < 0.05. Cefepime and tobramycin ± vancomycin were utilized most often.

For mortality prediction, time until first antibiotic in univariate analysis was nearly significant, *p* = 0.053, and the appropriate versus inappropriate antibiotic did not affect mortality. Regarding LOS, those treated with a single antibiotic had significantly increased LOS (*p* = 0.0089) and those treated with a CAP antibiotic had a shorter LOS (*p* = 0.049). Patients receiving clindamycin or piperacillin/tazobactam had a longer LOS (*p* = 0.043 and 0.038), respectively. There was poor correlation between LOS and time until first ABX or LOS and time until appropriate ABX with a correlation coefficient of -0.048 (*p* = 0.42) and -0.08 (*p* = 0.43), respectively.

Patients that died during initial hospitalization had a higher respiratory rate, glucose, blood urea nitrogen (BUN) and creatinine levels; lower GCS scores and hemoglobin level; more dyspnea on presentation; and increased incidence of mental status changes and intubations than those discharged alive. On step-down multivariable logistic regression analysis, increased heart rate, hematocrit and BUN, decreased temperature and hemoglobin, and presence of a mental status change were found to be the strongest predictors of death (Table 2). Patients who were intubated (19.57 ± 16.8 vs. 9.31 ± 6.6, *p* < 0.0001) and febrile (10.07 ± 9.6 vs. 11.58 ± 9, *p* < 0.012) had longer LOS. As age increases, LOS decreases (Spearman’s correlation coefficient -0.140, *p* = 0.017); and as respiratory rate and temperature increase, LOS increases (Spearman’s correlation coefficient 0.165 and 0.146, *p* = 0.006 and 0.015, respectively).

**Table 2:** Predictors of mortality in the ED

	**Parameter estimate**	***P *** **value**	**Odds ratio**	**95% Confidence interval**
Heart rate	0.0117	0.028	1.012	1.001-1.022
Temperature	-0.4184	0.0080	0.66	0.48-0.90
Mental status change	1.4054	0.0005	4.08	1.84-9.01
Hemoglobin	-1.1004	0.0042	0.33	0.16-0.71
Hematocrit	0.2916	0.016	1.34	1.06-1.70
BUN	0.0124	0.037	1.012	1.001-1.024

## Discussion

**Figure 3:**
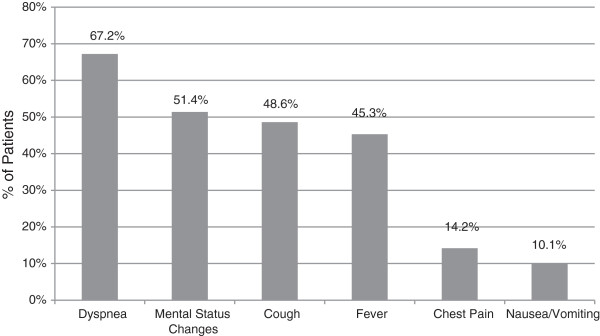
Symptoms on presentation to the ED.

**Figure 4:**
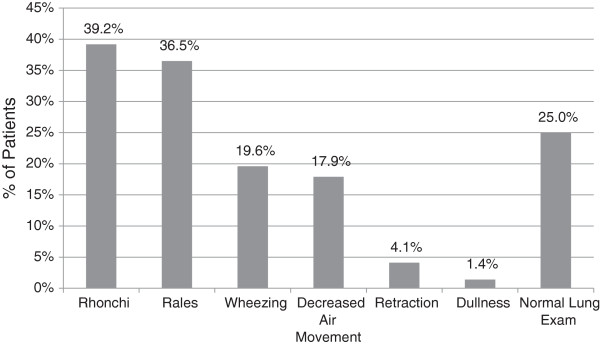
Physical examination findings on presentation to the ED.

This data set represents one of the largest studies of nursing home patients with pneumonia presenting to the ED. We found a surprisingly low prevalence of true-positive blood cultures (9%) and low yield and poor correlation in sputum cultures, high incidence of antibiotic pre-treatment at nursing homes prior to admission (27.7%), high hospital and 1-year mortality (16.2% and 24.2%) and low immunization rates (18.9%). There was a wide spectrum of pathogens grown in blood culture. Only two thirds of the patients had dyspnea as the presenting complaint and less than half had either cough or fever (Figure 3). On physical examination of the chest and lungs, about one fourth had no clinical findings consistent with pneumonia (Figure 4). Further, less than one fifth of chest x-rays were interpreted as possible pneumonia. These findings coupled with few recorded fevers, hypoxia and elevated WBC counts make the ED diagnosis of pneumonia very difficult and therefore affects the management (Tables 3 and 4).

**Table 3:** Vital signs on presentation to the ED

	**Mean (±SD)**
Systolic blood pressure	131.86 mmHg (SD ± 30.14)
Diastolic blood pressure	66.82 mmHg (SD ± 16.12)
Heart rate	92.38 beats per minute (SD ± 22.92)
Respiratory rate	24.74 breaths per minute (SD ± 6.97)
Temperature	37.35°C (SD ± 1.08)
Oxygen saturation	94.15% (SD ± 6.085)
Initial GCS	12.82 (SD ± 2.96)

**Table 4:** Laboratory values on presentation to the ED

	**Mean (±SD)**
WBC Count	13.8 × 10^9^/L (SD ± 12.3)
Hemoglobin Level	11.4 mg/dL (SD ± 2.0),
Hematocrit	34.9% (SD ± 6.4)
Platelet count	287 × 10^9^/L (SD ± 136.8)
Neutrophil count	10.7 × 10^9^/L (SD ± 6.8)
Lymphocyte count	1.6 × 10^9^/L (SD ± 5.0)
Blood glucose level	146.39 mg/dL (SD ± 78.1)
BUN	36.07 mg/dL (SD ± 26.4)
Serum sodium concentration	138.17 mmol/L (SD ± 13.8)
Serum creatinine level	1.7 mg/dL (SD ± 1.5)

Since the 2005 ATS/IDSA guidelines, there has been a decline in the number of appropriately treated NHAP patients, as recommended by these guidelines, but also a decrease in the number of patients who were treated as having CAP or treated with a single antibiotic.

There was a correlation between time to first ABX and mortality. Those patients that received only a single ABX had a significantly increased LOS. Clindamycin, piperacillin/tazobactam or CAP-directed antibiotics also affected LOS; however, co-linearity with severity of disease may be present.

The best early ED predictors of in-hospital mortality are decreased mental status and anemia. Elevated temperature and signs of dehydration were also predictive of in-hospital mortality. Hospital length of stay was related to age, elevated temperature, intubation and respiratory rate.

## Conclusions

In this data set of NHAP patients admitted through the ED, we found a surprisingly low prevalence of true-positive blood cultures and low yield and poor correlation in sputum cultures, high incidence of antibiotic pre-treatment at nursing homes prior to admission, high hospital mortality and low immunization rates. There was a wide spectrum of pathogens grown in blood culture. Only two thirds of the patients had dyspnea as the presenting complaint, and less than half had either cough or fever. On physical examination, about one fourth had no clinical findings consistent with pneumonia. Further, less than one fifth of chest x-rays were interpreted as possible pneumonia. In the light of these findings, we conclude that the patients presenting with pneumonia to the ED from nursing homes and long-term care facilities are a high-risk group with significant mortality and present unique diagnostic and treatment challenges to emergency physicians. Prospective, high-quality randomized controlled trials investigating empiric antibiotic therapy to determine the effect of appropriate regimen on clinical outcomes are needed to support the implementation of the ATS/IDSA guidelines.

## Competing interests

The authors declare that they have no competing interests.

## Authors’ contributions

SA performed the statistical analysis, interpreted the data and drafted the manuscript. NH contributed to data acquisition. CP interpreted the data and drafted the manuscript. PM and DR helped in data acquisition and analysis, and drafted the manuscript. RW drafted the manuscript. MZ and BON conceived the study, participated in its design and drafted the manuscript. All authors read and approved the final manuscript.

## Authors’ information

MZ is the Division Head of Infectious Diseases at Henry Ford Health System in Detroit, Michigan.

BON is the Chair of the Department of Emergency Medicine at Wayne State University and Specialist-in-Chief of Emergency Medicine at Detroit Medical Center in Detroit, Michigan.
